# Bridging the Gap: How High School Belonging and Innovative Behavior Retrospective Links Long-Term Pro-Environmental Behavior Through an Extended Theory of Planned Behavior Model

**DOI:** 10.3390/bs16071144

**Published:** 2026-07-08

**Authors:** Yaqiong He, Liufeng Xiao, Mengcheng Wang, Fen Ren

**Affiliations:** 1 School of Geography and Remote Sensing, Guangzhou University, Guangzhou 510006, China; geoheyq@gzhu.edu.cn (Y.H.); liufeng_xiao@e.gzhu.edu.cn (L.X.); 2 Department of Psychology, Guangzhou University, Guangzhou 510006, China; wmcheng2006@126.com; 3 School of Education and Psychology, University of Jinan, No. 336, West Nanxinzhuang Road, Jinan 250022, China

**Keywords:** theory of planned behavior, pro-environmental behavior, school belonging, pro-environmental innovative behavior

## Abstract

Environmental education is crucial for promoting sustainable behavior, yet maintaining engagement across educational transitions remains challenging. Drawing on the Theory of Planned Behavior (TPB), this study examined how high school experiences influence pro-environmental behavior (PEB) during the transition to university. We integrated sense of school belonging (SB) and pro-environmental innovative behavior (IB) as school-based antecedents to the TPB framework. A retrospective survey was conducted with 1879 first-year university students, and structural equation modeling (SEM) was used to test the hypothesized relationships. Results provided robust support for the integrated model. Consistent with TPB, attitudes, subjective norms, and perceived behavioral control significantly predicted pro-environmental intention, which subsequently predicted actual PEB. Both IB and SB emerged as significant predictors of these cognitive factors, with SB also promoting engagement in innovative activities. The model explained 72.4% of the variance in intention and 37.5% in actual behavior. Multigroup analysis revealed that while the structural model was stable across gender, certain pathway strengths varied. These findings offer empirical evidence for the retrospective associations of pro-environmental behavior, demonstrating that the recalled social–emotional and experiential contexts of high school are consistently related to university students’ current environmental actions. This study extends the TPB by highlighting the developmental role of school environments in fostering long-term environmental responsibility.

## 1. Introduction

In the face of escalating global climate change, environmental education has become an increasingly important component of school curricula (Monroe et al., 2019). However, a critical yet underexplored challenge concerns the continuity of students’ pro-environmental behavior (hereafter referred to as PEB) across educational stages. Although student engagement and extracurricular participation often decline during the transition from high school to university, it remains unclear whether PEB follows a similar trajectory, suggesting this period as a potential risk for behavioral change rather than a confirmed decline. These transitional disruptions may challenge the social and structural supports that sustain environmentally responsible actions; therefore, we characterize these as significant environmental shifts rather than absolute losses of support (Eccles et al., 1993; Symonds et al., 2026). As many environmental actions are embedded within school-based activities and social norms, changes in the educational environment may undermine the conditions that support the maintenance of PEB across this transition. Such declines may be especially pronounced during transitions involving changes in learning environments, peer networks, and institutional expectations. Crucially, these transitional disruptions can sever the social and structural supports that normally sustain environmentally responsible actions. When familiar peer networks and mandatory school norms are removed, students must rely on internalized values and prior competencies to maintain their PEB. Therefore, high school experiences that foster a sense of belonging and encourage participation in innovative environmental practices may have lasting implications for students’ pro-environmental intentions beyond secondary education. First, school belonging provides a vital socio-emotional foundation. While educational transitions disrupt existing peer networks, a strong prior connection to the high school community facilitates the deep internalization of collective values (Wild & Schulze Heuling, 2024). Once environmental responsibility is internalized through a sense of belonging, it functions as a stable personal value that buffers against the loss of peer support during the university transition. Second, shifting institutional expectations mean university students face novel, unstructured environmental challenges with less direct supervision. In this context, students who previously engaged in pro-environmental innovative behavior have likely built strong self-efficacy and independent problem-solving skills (Otto et al., 2021). These mastery experiences equip them to navigate new institutional expectations and sustain pro-environmental actions without relying on rigid high school mandates.

Despite growing attention to environmental education, existing studies have largely focused on macro-level policies or cross-sectional outcomes, leaving the developmental processes that support sustained behavioral engagement insufficiently understood (Kollmuss & Agyeman, 2002). In particular, school-specific experiences, such as students’ sense of belonging and opportunities to engage in innovative environmental practices, may play an important role in shaping long-term behavioral patterns, but remain relatively understudied (Wild & Schulze Heuling, 2024; Otto et al., 2021). To address this gap, the present study examines how high school experiences retrospective links psychological processes that, in turn, influence pro-environmental behavior in university. It further explores how early school experiences contribute to the development of enduring environmental identities.

The Theory of Planned Behavior (TPB; Ajzen, 1991) provides a widely used framework for understanding how cognitive determinants translate into action. According to the TPB, behavioral intention is retrospective linked by three key antecedents: attitudes toward the behavior, subjective norms, and perceived behavioral control. Furthermore, the model explicitly posits that perceived behavioral control can also directly predict actual behavior, independent of intention, particularly when individuals face structural or practical barriers. While this framework has been successfully applied to explain pro-environmental behavior across diverse stable contexts (Bamberg & Möser, 2007; Tran et al., 2025; Prinzing & Laffan, 2024), its focus on proximal rational cognitions presents a limitation when addressing behavioral continuity across dynamic life changes.

Growing evidence suggests that students’ PEB often weakens during major educational transitions, such as the move from high school to university (Benner et al., 2016; Eccles et al., 1993; Symonds et al., 2026). This decline highlights a boundary condition of the traditional TPB framework: it relies heavily on rational cognitive evaluations within a stable context, and therefore does not sufficiently account for the socio-emotional disruptions and the loss of familiar contextual anchors that occur during major educational transitions. Because a purely cognitive model struggles to explain why established environmental intentions might decay when students are uprooted from their supportive environments, there is a critical need to extend the TPB. Incorporating past socio-emotional experiences (e.g., prior school belonging) and contextualized behavioral engagement (e.g., innovative environmental practices) into the TPB is essential for understanding how behavioral continuity is sustained over time despite environmental disruptions.

One important social–emotional factor is school belonging, defined as students’ sense of acceptance and connection within their educational environment (Osterman, 2000). A strong sense of belonging fosters identification with school values and promotes responsibility toward collective goals (Chrobak, 2024). Importantly, in contemporary educational contexts, environmental education and sustainability practices have become core components of the institutional ethos (Monroe et al., 2019). When schools emphasize collective environmental responsibility, a high sense of school belonging fosters organizational identification, which makes students more receptive to the specific environmental sustainability goals promoted by their institution, thereby facilitating the internalization of these norms. Consequently, when students feel deeply connected to their high school community, they are more likely to adopt the school’s pro-environmental values, thereby reporting stronger pro-environmental attitudes and perceived social expectations even after transitioning to university.

In addition to social–emotional factors, opportunities for active engagement in pro-environmental innovative practices during high school also play a foundational role. Pro-environmental innovative behavior refers to students’ efforts to generate or implement novel ideas aimed at addressing environmental challenges (De Jong & Den Hartog, 2010). Although environmental attitudes are often regarded as antecedents of innovative behavior, participation in innovative environmental practices may likewise retrospectively link later environmental beliefs, especially as students adapt to new educational contexts. According to social cognitive theory (Bandura, 2001), direct engagement in problem-solving provides critical “mastery experiences.” When students actively participate in designing or implementing environmental innovations during high school, they accumulate mastery experiences that specifically build their perceived behavioral control. Furthermore, public engagement in such innovative activities often elicits peer feedback and reinforces the visibility of environmental issues, which retrospectively strengthens their current pro-environmental attitudes and subjective norms in a new university setting.

Building on the TPB, the present study examines how high school experiences contribute to the development of pro-environmental behavior during students’ transition to university. In the present study, sense of school belonging and pro-environmental innovative behavior are considered as two school-based experiences that may influence students’ attitudes, perceived norms, and sense of control toward environmental behavior. Sense of school belonging is hypothesized to influence pro-environmental behavior indirectly by strengthening attitudes and subjective norms, whereas pro-environmental innovative behavior is expected to enhance pro-environmental attitudes, subjective norms, and perceived behavioral control through opportunities for active engagement and mastery experiences.

To examine these relationships, the present study used retrospective reports from a large sample of first-year university students, and employed structural equation modeling to test how high school experiences are associated with pro-environmental behavior at the university level. By focusing on the transition from high school to university, the study sought to understand whether PEB developed during secondary education continues to influence students’ behavior in later educational contexts.

By incorporating school belonging and pro-environmental innovative behavior into the TPB framework, this study provides a more integrated account of how social–emotional and behavioral experiences contribute to sustained pro-environmental behavior. The findings are expected to offer theoretical insight into how school experiences retrospectively link long-term PEB, while also informing school environmental design that encourages continued participation in environmental practices across educational stages.

Consistent with the TPB, attitudes, subjective norms, and perceived behavioral control are regarded as key determinants of behavioral intention, which in turn predicts actual behavior. In the context of pro-environmental behavior, students who hold favorable environmental attitudes, perceive stronger social expectations, and feel capable of performing environmental actions are more likely to form intention to engage in such behaviors. Indeed, behavioral intention has been widely identified as the most proximal predictor of actual behavior. Formulated as directional alternative hypotheses predicting positive associations between the specified constructs, the following hypotheses were proposed:

**H1a.**  *Students’ pro-environmental attitudes will be positively associated with their intention to engage in pro-environmental behavior.*

**H1b.**  *Students’ subjective norms will be positively associated with their intention to engage in pro-environmental behavior.*

**H1c.**  *Students’ perceived behavioral control will be positively associated with their intention to engage in pro-environmental behavior.*

**H1d.**  *Students’ intention to engage in pro-environmental behavior will be positively associated with their actual pro-environmental behavior.*

Building on the extended TPB framework proposed in this study, pro-environmental innovative behavior was expected to function as an important behavioral precursor that retrospectively links key cognitive components of environmental action. Engaging in innovative environmental practices may expose students to new ideas and reinforce environmental awareness. Crucially, according to social cognitive theory (Bandura, 2001), directly engaging in problem-solving serves as a “mastery experience” that enhances students’ perceived behavioral control and confidence in addressing environmental challenges. Furthermore, engaging in innovative environmental practices often involves public visibility and peer collaboration. When students actively propose or implement novel environmental solutions, their actions elicit social feedback and reinforce their public commitment to environmentalism. This heightened visibility makes them more sensitive to the pro-environmental expectations of their peer group, thereby strengthening their subjective norms (Vesely et al., 2021). Accordingly, the following hypotheses were proposed:

**H2a.**  *Students’ pro-environmental innovative behavior will be positively associated with their pro-environmental attitudes.*

**H2b.**  *Students’ pro-environmental innovative behavior will be positively associated with their subjective norms.*

**H2c.**  *Students’ pro-environmental innovative behavior will be positively associated with their perceived behavioral control.*

In addition to behavioral engagement, social–emotional experiences within school environments may also influence environmental decision-making. School belonging reflects students’ sense of acceptance and identification with their school community, which may promote the internalization of shared values and behavioral expectations. When students feel connected to their school environment, they may be more likely to adopt environmentally responsible values and perceive stronger social norms regarding environmental protection. Furthermore, beyond the internalization of values, school belonging also fosters a climate of psychological safety. When students feel accepted and supported by their school community, they perceive lower interpersonal risks when facing challenges (Wentzel, 1998). This supportive environment boosts their self-efficacy and confidence in their ability to enact change, thus enhancing their perceived behavioral control. Similarly, psychological safety is a critical prerequisite for innovation; students who feel they ‘belong’ are more willing to take the intellectual and social risks required to engage in pro-environmental innovative behavior. Therefore, the following hypotheses were proposed:

**H3a.**  *Students’ sense of school belonging will be positively associated with their pro-environmental attitudes.*

**H3b.**  *Students’ sense of school belonging will be positively associated with their subjective norms.*

**H3c.**  *Students’ sense of school belonging will be positively associated with their perceived behavioral control.*

**H3d.**  *Students’ sense of school belonging will be positively associated with their pro-environmental innovative behavior.*

Finally, previous research has identified gender differences in environmental attitudes and behavior, with female students typically reporting stronger environmental concern and engagement than their male counterparts (McCright & Dunlap, 2015; Zelezny et al., 2000). Whether the pathways linking high school experiences to later TPB-related cognitions differ by gender, however, remains largely unexplored. Accordingly, we conducted an exploratory multigroup analysis to examine potential gender differences in the structural paths of the model, without proposing specific directional hypotheses.

**Figure 1:**
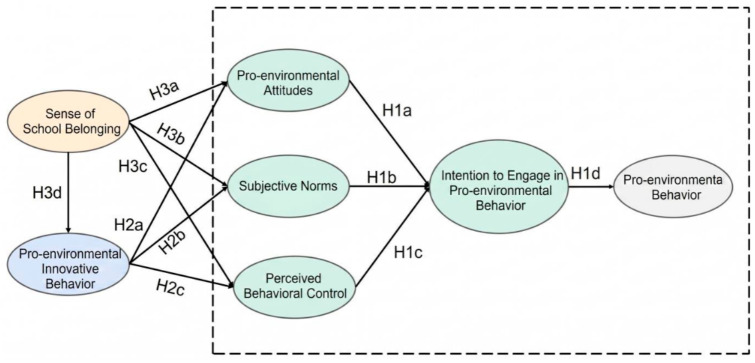
Conceptual model of the proposed relationships among study variables.

Based on the preceding theoretical framework and hypotheses, a conceptual model was developed to illustrate the proposed relationships among high school experiences, psychological determinants, and students’ pro-environmental behavior. The hypothesized model is presented in Figure 1.

## 2. Methods

### 2.1. Participants

A total of 1879 first-year university students were recruited from several higher education institutions in China. Participants were invited to participate through faculty-wide announcements, and eligibility was restricted to current first-year undergraduate students. Exclusion criteria were applied to ensure data quality: specifically, surveys were excluded if respondents failed to complete all mandatory sections or if the total survey completion time was less than 3 min, indicating insufficient engagement. The adequacy of this sample size was justified based on the parameter-to-sample-size ratio and established guidelines for structural equation modeling (SEM). According to Hair et al. (2019), complex structural models require a minimum sample size of 500, and an ideal observation-to-parameter ratio should be at least 10:1 to ensure model stability. Given the complexity of our integrated model, the final sample of 1879 effectively exceeds these stringent thresholds, providing robust statistical power to test the hypothesized multi-path relationships. Data were collected during the students’ first semester to minimize retrospective bias regarding their high school experiences. Among the participants, 71.8% were female (*n* = 1350), 27.7% were male (*n* = 520), and 0.5% did not report their gender (*n* = 9). The participants ranged in age from 18 to 23 years (*M* = 18.29, *SD* = 0.64). The sample represented a diverse range of academic disciplines, including humanities, social sciences, natural sciences, and engineering. All 1879 cases met the predefined criteria for completeness and response quality after initial screening, thus being included in the final analysis.

### 2.2. Procedures

The study employed a retrospective survey design to examine the long-term associations of secondary school experiences on students’ current pro-PEB. Data were collected in 2023 in a classroom setting under standardized conditions, supervised by trained research assistants to ensure procedural consistency across sessions. Prior to participation, students were informed about the purpose of the study and assured that their responses would remain anonymous and confidential. Participation was voluntary, and written informed consent was obtained from all participants before the survey administration. Participants completed a structured multi-part questionnaire that required approximately 20 to 25 min to finish. The questionnaire asked participants to retrospectively report their high school experiences (e.g., sense of school belonging and pro-environmental innovative behavior) and to indicate their current pro-environmental attitudes, perceived behavioral control, subjective norms, behavioral intention, and pro-environmental behaviors. To enhance data quality, research assistants were available to clarify item instructions and terminology when necessary without providing guidance that could influence participants’ responses. All study procedures were reviewed and approved by the Institutional Review Board of the University of Jinan (Ethics Code: UJN2023-47).

### 2.3. Measures

All variables in this study were measured using established scales adapted from prior research and translated into Chinese to suit the context of the target population. A 5-point Likert scale was employed for all items, ranging from 1 (“very inconsistent/strongly disagree”) to 5 (“fully consistent/strongly agree”).

#### 2.3.1. Pro-Environmental Behavior

The Pro-environmental Behavior Scale, adapted from Kaiser and Wilson (2000), comprised 14 items (e.g., “I will take the initiative to serve as an environmental volunteer in a leisure place”) measuring students’ participation in environmental protection within public and leisure spaces. The scale demonstrated good internal consistency in the current study, with a Cronbach’s α of 0.785.

#### 2.3.2. Pro-Environmental Attitudes

Students’ attitude toward environmental protection was measured using a five-item scale adapted from the New Ecological Paradigm scale (Dunlap et al., 2000). A sample item is “Protecting the environment is wise.” This dimension evaluated the perceived value and emotional satisfaction derived from environmental actions. In the present study, the scale demonstrated acceptable internal consistency with a Cronbach’s α of 0.714.

#### 2.3.3. Intention to Engage in Pro-Environmental Behavior

Based on the Reasoned Action Approach (Fishbein & Ajzen, 2011), intention to engage in pro-environmental behavior (or “pro-environmental intention”) was assessed using a six-item scale. These items reflected students’ willingness to acquire environmental knowledge and participate in conservation projects (e.g., “I will learn the knowledge of environmental protection”). The scale demonstrated excellent internal reliability in this study, with a Cronbach’s α of 0.835.

#### 2.3.4. Perceived Behavioral Control

Following the TPB framework (Ajzen, 1991) and recommendations for tailoring measures to specific behavioral contexts (Ajzen, 2002), perceived behavioral control was assessed using an adapted three-item scale. Rather than removing items to reduce survey length, these three items were selected through a theoretical appraisal process by a panel of experts to ensure the scale captured the essential theoretical components of perceived behavioral control within the specific context of campus environmental protection. The term ‘adapted’ refers to the translation into Chinese and minor rephrasing to maintain conceptual equivalence with the original TPB framework. This measurement approach reflects the practice of tailoring TPB scales to specific contexts to enhance their predictive validity, as recommended by Ajzen (2002). These specific three items were selected from the original item pool for their high face validity and contextual relevance to students’ perceived capability within a campus environment (e.g., “My environmental protection action has a substantial effect on campus environmental protection”). As noted previously, the Cronbach’s α for this scale was 0.653, which is considered acceptable for a short three-item subscale in behavioral research (DeVellis & Thorpe, 2021).

#### 2.3.5. Subjective Norms

Subjective norms were measured using a three-item scale derived from Ajzen’s (1991) theoretical framework. The scale evaluated the extent to which students perceived social pressures from important referents to engage in environmental protection, with items such as, “Those who are important to me take the initiative to protect the campus environment.” The instrument exhibited good reliability, evidenced by a Cronbach’s α of 0.792.

#### 2.3.6. Pro-Environmental Innovative Behavior

Drawing on the conceptualization of individual innovation by Scott and Bruce (1994), pro-environmental innovative behavior was measured using a nine-item scale. This instrument assessed students’ proactive engagement in identifying and promoting creative solutions to environmental issues (e.g., “When I encounter environmental problems, I take the initiative to find innovative solutions”). In the current study, the scale exhibited high internal reliability, with a Cronbach’s α of 0.860.

#### 2.3.7. Sense of School Belonging

Students’ sense of school belonging was assessed using an 18-item scale based on the work of Goodenow (1993). The scale captured multiple facets of school membership, including emotional attachment and social acceptance by teachers and peers. A sample item is, “I feel like I’m completely part of my high school alma mater.” The internal consistency for this dimension was robust (α = 0.794).

### 2.4. Data Analysis

Data analysis was conducted using SPSS 26.0 and Mplus 8.3. Prior to the main analyses, data screening was performed. Given the structured survey administration, participants were required to complete all items; thus, there were no missing data in the final dataset. Descriptive statistics, Pearson correlations, reliability analyses (Cronbach’s α), and Harman’s single-factor test for common method bias were computed using SPSS. Subsequently, structural equation modeling (SEM) and multigroup analyses were performed using Mplus with the Maximum Likelihood (ML) estimation method. Following a two-step approach, we first evaluated the measurement model via Confirmatory Factor Analysis (CFA) to establish construct validity and discriminant validity. Next, the structural model was tested to examine the hypothesized relationships. The adequacy of the model fit was evaluated using standard indices (Hair et al., 2019): the ratio of chi-square to degrees of freedom (χ^2^/*df*, where values < 5 indicate acceptable fit), the Root Mean Square Error of Approximation (RMSEA, values < 0.08), the Comparative Fit Index (CFI, values > 0.90), and the Tucker–Lewis Index (TLI, values > 0.90). Finally, a multigroup SEM was conducted to test for measurement invariance and compare structural path differences across genders.

## 3. Results

### 3.1. Common Method Bias Assessment

Given that all data in this study were collected via self-report questionnaires during a single session, common method variance (CMV) was assessed using Harman’s single-factor test (Podsakoff et al., 2003) prior to the structural modeling. All 55 items from the measurement scales were subjected to an unrotated principal component analysis. The results revealed that the first unrotated factor accounted for 20.84% of the total variance, which is well below the strictly recommended threshold of 40%. This indicates that no single dominant factor emerged to explain the majority of the variance, suggesting that common method bias did not severely inflate the relationships among the variables in the current study.

### 3.2. Descriptive Statistics and Correlations

Table 1 presents the means (*M*), standard deviations (*SD*), and Pearson correlation coefficients (*r*) among the observed composite scores of all study variables. The mean scores for pro-environmental attitudes (*M* = 4.31, *SD* = 0.65) and intention (*M* = 4.12, *SD* = 0.72) were relatively high, suggesting that participants generally held positive orientations toward environmental conservation. Pro-environmental behavior also showed a moderate-to-high level of engagement (*M* = 3.92, *SD* = 0.68). As expected, correlation analysis revealed that all variables were significantly and positively intercorrelated (*p* < 0.01). Specifically, pro-environmental intention exhibited strong positive correlations with attitude (*r* = 0.83), subjective norms (*r* = 0.40), and perceived behavioral control (*r* = 0.51). Furthermore, sense of school belonging and pro-environmental innovative behavior were significantly associated with both the psychological variables and actual behaviors. A detailed correlation matrix is provided in Table 1.

### 3.3. Measurement Model

Before testing the structural relationships, the measurement model was evaluated for reliability and validity. Confirmatory factor analysis results indicated that the measurement model fitted the data well: χ^2^/*df* = 2.65, RMSEA = 0.028, CFI = 0.975, and TLI = 0.971. To ensure measurement quality, standardized factor loadings, composite reliability (CR), and average variance extracted (AVE) were examined (see Table 2). All standardized factor loadings were significant (*p* < 0.001). The CR values for all constructs ranged from 0.844 to 0.923, which safely exceed the recommended 0.70 benchmark. Following the refinement of the measurement model by removing items with standardized factor loadings below 0.40, all constructs exhibited satisfactory convergent validity, with AVE values exceeding the 0.50 threshold (ranging from 0.481 to 0.712). While the AVE values for the majority of constructs exceeded the 0.50 threshold (ranging up to 0.712), the AVEs for some broad behavioral scales (e.g., pro-environmental behavior) were marginally below 0.50. However, because their corresponding CR values were well above the 0.60 threshold, the convergent validity of these constructs is still considered adequate (Fornell & Larcker, 1981). Furthermore, discriminant validity was assessed using the Heterotrait–Monotrait (HTMT) ratio of correlations. The HTMT value between environmental attitude and pro-environmental intention was 0.58, which is well below the conservative threshold of 0.90 (Henseler et al., 2015). This confirms that despite their close theoretical relationship within the TPB framework, these dimensions represent empirically distinguishable constructs in our measurement model, thereby mitigating concerns regarding conceptual overlap.

### 3.4. Structural Model and Hypothesis Testing

A structural equation modeling approach was employed to test the hypothesized paths among sense of school belonging, TPB components (i.e., pro-environmental attitudes, subjective norms, and perceived behavioral control), pro-environmental innovative behavior, intention, and actual behavior. The structural model yielded a good fit to the observed data: χ^2^/*df* = 2.88, RMSEA = 0.031, CFI = 0.962, and TLI = 0.958. As illustrated in Table 3, all hypothesized paths were statistically significant. It should be noted that we initially tested the direct path from perceived behavioral control to PEB; however, the path coefficient was statistically non-significant (β = 0.04, *p* > 0.05). Consequently, this path was omitted from the final structural model to adhere to the principle of parsimony and improve model fit. Regarding the antecedents of behavioral intention (H1a–H1c), pro-environmental attitudes (β = 0.921, *p* < 0.001), subjective norms (β = 0.305, *p* < 0.001), and perceived behavioral control (β = 0.514, *p* < 0.001), each exerted a significant positive influence on pro-environmental intention. In turn, pro-environmental intention was a robust predictor of actual pro-environmental behavior (β = 0.681, *p* < 0.001), providing strong support for H1d. The model also identified pro-environmental innovative behavior as an important correlate within the TPB framework. Specifically, pro-environmental innovative behavior was positively associated with pro-environmental attitudes (β = 0.219), subjective norms (β = 0.486), and perceived behavior control (β = 0.271), with all paths significant at the *p*s < 0.001 level, supporting H2a, H2b, and H2c. Finally, sense of school belonging functioned as a distal antecedent in the model. Sense of school belonging significantly and positively predicted pro-environmental attitudes (β = 0.205), subjective norms (β = 0.402), perceived behavioral control (β = 0.208), and pro-environmental innovative behavior (β = 0.379), supporting H3a, H3b, H3c, and H3d. Overall, the model accounted for 68.1% of the variance in pro-environmental intentions and 26.5% of the variance in pro-environmental behavior.

### 3.5. Measurement Invariance and Multigroup Analysis by Gender

Prior to comparing the structural paths between male and female students, measurement invariance was established to ensure that the latent constructs held the same meaning across genders (Putnick & Bornstein, 2016). We sequentially tested configural (unconstrained), metric (constrained factor loadings), and scalar (constrained item intercepts) invariance models. Specifically, the baseline configural model yielded an acceptable fit (χ^2^/*df* = 3.48, CFI = 0.945, RMSEA = 0.037). Constraining the factor loadings to be equal across genders for metric invariance did not significantly degrade the model fit (ΔCFI = 0.004, ΔRMSEA = 0.001). Further constraining the item intercepts for scalar invariance also resulted in changes well within the recommended thresholds (ΔCFI = 0.006, ΔRMSEA = 0.002). Because the fit decrements at each increasingly restrictive step were strictly bounded (ΔCFI < 0.010 and ΔRMSEA < 0.015), strong scalar invariance was confirmed across genders, justifying the subsequent multigroup structural comparisons.

To evaluate the structural invariance of the proposed model, a multigroup analysis was conducted to compare the path coefficients between male and female students. As summarized in Table 4, the model demonstrated high stability across groups, with all 11 hypothesized paths remaining statistically significant for both genders (*p*s < 0.01). Interesting variations were observed in the magnitude of certain effects. For male students, the influence of pro-environmental attitudes on intention (H1a: β_Male_ = 0.945 vs. β_Female_ = 0.885) and the subsequent translation of intention into behavior (H1d: β_Male_ = 0.705 vs. β_Female_ = 0.655) were noticeably stronger than for their female counterparts. This suggests that for males, personal values and internal drive may play a more dominant role in governing environmental actions. Furthermore, sense of school belonging appeared to have a more pronounced impact on male students’ psychological precursors, particularly on their pro-environmental attitudes (H3a: β_Male_ = 0.225 vs. β_Female_ = 0.180) and perceived behavioral control (H3c: β_Male_ = 0.225 vs. β_Female_ = 0.190). These results indicate that a positive school environment might be a more critical catalyst for developing environmental agency among male adolescents than for females. Despite these differences in magnitude, the overall structural relationships were consistent, confirming the generalizability of the integrated TPB and school belonging framework.

## 4. Discussion

The present study aimed to extend the Theory of Planned Behavior (TPB) by integrating pro-environmental innovative behavior and sense of school belonging into a unified framework to explain students’ pro-environmental behavior. Overall, the structural equation modeling results reveal significant cross-sectional associations among these variables. Rather than demonstrating definitive developmental causality, our findings provide robust statistical support for the theoretical proposition that recalled positive social–emotional experiences in high school are closely linked to students’ current cognitive readiness and behavioral intentions. Within this retrospective design, all proposed hypotheses (H1a–H3d) were statistically supported. The model demonstrated substantial explanatory power, accounting for 72.4% of the variance in pro-environmental intention and 37.5% of the variance in actual pro-environmental behavior. This suggests that the inclusion of prior behavioral and socio-emotional variables significantly enhances the explanatory capacity of traditional TPB models. However, it is essential to interpret these findings within the boundaries of our methodology. Our study adopts a cross-sectional, retrospective design rather than a longitudinal approach. Therefore, we do not claim to observe definitive developmental causality or longitudinal stability; rather, we propose that the observed associations indicate the potential psychological significance of recalled experiences in understanding current environmental orientations. Ultimately, these findings contribute to a growing body of literature indicating that environmental behavior is consistently associated not only with rational cognitive processes but also with experiential and contextual influences (Ajzen, 1991; Bamberg & Möser, 2007).

### 4.1. TPB Relationships and Behavioral Mechanisms (H1a–H1d)

Consistent with the foundational assumptions of the TPB (Ajzen, 1991), pro-environmental attitude, subjective norms, and perceived behavioral control each exerted significant positive effects on students’ pro-environmental intention. These findings provide strong support for H1a–H1c and align with prior meta-analytic evidence demonstrating that these three constructs are reliable predictors of pro-environmental intention across diverse contexts (Bamberg & Möser, 2007). Among the three predictors, perceived behavioral control emerged as the strongest predictor of intention, highlighting the importance of students’ perceived capability and confidence in engaging in environmental action. This finding is consistent with previous research suggesting that individuals who believe they possess sufficient skills and resources are more likely to form strong behavioral intentions (Bandura & Wessels, 1997; Ajzen, 2002). In educational settings, this underscores the importance of equipping students with practical knowledge and actionable strategies that enhance their sense of competence in environmental protection. Furthermore, pro-environmental intention demonstrated a robust positive association with actual pro-environmental behavior, providing strong support for H1d. The magnitude of this relationship confirms the TPB assumption that intention functions as the most proximal determinant of behavior. This finding is consistent with earlier studies showing that intention reliably predicts environmentally responsible behavior, particularly when individuals perceive behavioral control and social support (Kaiser et al., 2005; Ajzen et al., 2018). Notably, the strong association between pro-environmental attitudes and intention further emphasizes the importance of cultivating positive environmental values during adolescence, a critical developmental stage during which environmental beliefs and behavioral norms become internalized (Evans et al., 2018).

### 4.2. The Role of Pro-Environmental Innovative Behavior (H2a–H2c)

Beyond traditional TPB components, the present study identified pro-environmental innovative behavior as an important behavioral precursor influencing environmental cognition. Specifically, innovative behavior significantly predicted pro-environmental attitudes, subjective norms, and perceived behavioral control, supporting H2a–H2c. Among these pathways, the strongest association was observed between innovative behavior and subjective norms. This suggests that engaging in innovative environmental activities may increase students’ visibility within peer groups and strengthen their sensitivity to shared expectations regarding environmental responsibility. Such experiences may reinforce collective environmental values and promote norm-based motivation (Cialdini et al., 1990; Vesely et al., 2021). Recent evidence suggests that in collective settings like schools, perceived group norms significantly bolster individual commitment to sustainability (Fornara et al., 2020). These findings extend previous research indicating that innovative behavior fosters not only creative problem-solving but also social learning processes that enhance environmental awareness (Scott & Bruce, 1994; West & Farr, 1990). Recent evidence further supports this link, suggesting that engaging in pro-environmental innovation facilitates dynamic knowledge exchange and collaborative learning among peers (Sahoo et al., 2023). Such innovative practices serve as a catalyst for cognitive restructuring, allowing individuals to internalize complex ecological issues through active experimentation and social interaction (Treffinger et al., 2021). When students actively participate in designing or implementing environmental solutions, they may experience a heightened sense of ownership and responsibility, which strengthens their environmental beliefs and perceived efficacy. Furthermore, the positive relationship between innovative behavior and perceived behavioral control suggests that experiential engagement plays a crucial role in building self-efficacy. According to social cognitive theory (Bandura, 2001), mastery experiences are among the most powerful sources of efficacy beliefs. Thus, engaging students in hands-on environmental innovation may represent an effective pathway for strengthening their confidence in environmental action.

### 4.3. The Influence of School Belonging as a Distal Contextual Factor (H3a–H3d)

The present findings also highlight sense of school belonging as a significant distal antecedent influencing both cognitive and behavioral aspects of PEB. Students who reported stronger school belonging were more likely to exhibit positive environmental attitudes, perceive stronger subjective norms, experience greater perceived behavioral control, and engage more actively in pro-environmental innovative behavior, providing support for H3a–H3d. Notably, the strongest effect of school belonging was observed on subjective norms. This finding suggests that students who feel connected to their school community may be more sensitive to collective expectations and shared environmental values. These results are consistent with a growing body of recent international research, which emphasizes that school belonging acts as a psychological “secure base” for internalization of social values in diverse cultural contexts (e.g., Allen et al., 2021). Such results are consistent with research indicating that belongingness strengthens conformity to group norms and promotes prosocial behavior (Goodenow, 1993; Osterman, 2000). The relationship between school belonging and innovative behavior further indicates that emotionally supportive school environments may encourage initiative and creativity. When students feel psychologically safe and socially accepted, they are more likely to take risks and explore novel solutions to environmental problems (Wentzel, 1998). This highlights the importance of fostering inclusive school climates that encourage both emotional connection and intellectual exploration. These findings support ecological perspectives suggesting that environmental behavior develops within relational contexts and is influenced by social identity processes (Bronfenbrenner, 1979; Ryan & Deci, 2000).

### 4.4. Gender Differences in Structural Relationships

The multigroup analysis demonstrated that the overall structure of the proposed model remained stable across gender, indicating strong generalizability of the integrated framework. However, meaningful differences were observed in the magnitude of several pathways. In particular, pro-environmental attitudes exerted a stronger influence on pro-environmental intention among male students compared to females. Additionally, the pathway from intention to behavior was more pronounced among male students, suggesting that once pro-environmental intention is formed, male adolescents may be more likely to translate these motivations into concrete actions. This finding supports previous research indicating that gender differences may emerge in motivational processes underlying PEB (Zelezny et al., 2000; Zelezny & Schultz, 2000). Furthermore, sense of school belonging demonstrated stronger effects on pro-environmental attitudes and perceived behavioral control among male students (Krumm, 2024). These results suggest that male students may rely more heavily on school-based emotional support in developing environmental competence and confidence. In contrast, female students may draw upon a broader range of interpersonal or contextual influences. Collectively, these gender-specific patterns highlight that while the TPB framework is structurally robust, the psychological pathways to environmental engagement are nuanced by gender socialization. Our findings suggest that environmental education programs should not adopt a “one-size-fits-all” approach; instead, they might maximize effectiveness by fostering school-based emotional support for male students to build self-efficacy, while leveraging the diverse, broader social–relational networks that female students typically navigate to promote their environmental action. Despite these differences, the consistency of statistically significant paths across both groups confirms the overall structural stability of the proposed model.

### 4.5. Theoretical Contributions

The present study makes several important theoretical contributions. First, this research extends the TPB framework by incorporating pro-environmental innovative behavior as a meaningful antecedent influencing cognitive determinants of intention. By demonstrating that innovative behavior retrospective links attitudes, subjective norms, and perceived behavioral control, the study provides a more dynamic interpretation of behavioral formation processes (Ajzen, 1991; Scott & Bruce, 1994). Second, the study highlights the role of socio-emotional context, particularly school belonging, as a foundational factor influencing PEB (Ciocîrlan et al., 2025). This expands prior TPB-based research by integrating relational and contextual variables into behavioral prediction models (Goodenow, 1993; Osterman, 2000). Third, the high explanatory power of the model suggests that integrating cognitive, behavioral, and emotional components offers a comprehensive framework for understanding adolescent PEB. This integrated perspective responds to recent calls for multi-dimensional approaches to environmental education research (Evans et al., 2018).

### 4.6. Practical Implications

The findings offer specific implications for educational practice and environmental policy, tailored to the structural paths identified in our model. First, given the significant effect of perceived behavioral control (PBC) on environmental intention and behavior, educational interventions should prioritize strengthening students’ sense of agency and competence. Instead of general awareness-raising, schools should implement project-based learning and student-led sustainability initiatives that provide students with tangible opportunities to overcome environmental challenges. Such experiences are critical for building the perceived competence necessary to translate environmental intentions into action (Bandura, 2001). Second, our model highlights the role of pro-environmental innovative behavior as a primary antecedent to cognitive readiness. Educators should therefore integrate “innovation-oriented” tasks—such as designing creative solutions for local environmental issues—into the curriculum. This strategy effectively fosters the environmental attitudes and norms that precede formal intentions, moving beyond passive learning toward active identity construction. Third, the positive influence of sense of school belonging on environmental attitudes and perceived control suggests that the social–emotional climate of an institution is a foundational environmental strategy. Institutions should invest in cultivating inclusive and collaborative cultures, as a strong sense of belonging appears to create a secure psychological base that enhances students’ receptivity to environmental values (Osterman, 2000). Finally, our exploratory gender analysis suggests that gender-sensitive pedagogical approaches are warranted. Since male students showed a stronger reliance on school-based support for developing environmental competence, interventions in male-dominated academic contexts should emphasize structural and collaborative support systems. Conversely, for female students, who may leverage broader interpersonal networks, interventions could focus on enhancing the social–relational aspects of environmental engagement. By aligning pedagogical strategies with these distinct structural pathways, schools can move toward more precise and effective environmental education.

### 4.7. Limitations and Future Research

Despite the contributions of this study, several limitations should be acknowledged. First, the cross-sectional and retrospective survey design relied on participants’ recall of high school experiences. Although collecting data during the first semester of university may reduce recall bias, it does not completely remove it. More importantly, while our design effectively demonstrates robust associations between recalled high school experiences and current pro-environmental outcomes, it cannot definitively establish long-term causal influence. The relationships identified should be interpreted as structural associations rather than causal pathways. Specifically, as participants evaluated their past experiences through the lens of their current university adaptation, their reports may be subject to “consistency bias,” where current attitudes influence the recollection of past emotions (Conway & Ross, 1984). While we attempted to mitigate this by focusing on stable socio-emotional constructs, future research should utilize longitudinal designs to minimize retrospective subjectivity and capture developmental trajectories more accurately. Second, the reliance on self-report measures may have increased the risk of common method bias; thus, future research should incorporate behavioral observations or multi-source data to enhance measurement validity. Third, the sample was drawn from a specific educational context, which may limit generalizability. Replication across diverse cultural and institutional contexts would strengthen the robustness of the proposed model. Finally, future studies may explore additional mediating mechanisms, such as environmental identity or moral obligation, to further elucidate the pathways linking school belonging to environmental outcomes.

Nevertheless, the substantial consistency observed in our model suggests that these recalled experiences remain psychologically salient predictors of students’ current environmental orientations, regardless of potential memory fluctuations.

## Figures and Tables

**Table 1:** Descriptive statistics and intercorrelations of variables.

Variable	*M*	*SD*	1	2	3	4	5	6	7
1. PEA	4.31	0.65	1						
2. SN	3.28	0.81	0.42 **	1					
3. PBC	3.95	0.74	0.46 **	0.44 **	1				
4. IPB	4.12	0.72	0.83 **	0.40 **	0.51 **	1			
5. IB	3.76	0.82	0.15 **	0.45 **	0.34 **	0.21 **	1		
6. SB	3.58	0.68	0.18 **	0.35 **	0.27 **	0.16 **	0.23 **	1	
7. PEB	3.92	0.68	0.41 **	0.38 **	0.35 **	0.50 **	0.36 **	0.25 **	1

Note. PEA = Pro-environmental attitudes; SN = Subjective norms; PBC = Perceived behavioral control; IB = Pro-environmental innovative behavior; IPB = Intention to engage in pro-environmental behavior; SB = Sense of school belonging; PEB = Pro-environmental behavior; ** *p* < 0.01.

**Table 2:** Reliability and convergent validity of the measurement model.

Construct	Abbreviation	Standardized Factor Loadings	Composite Reliability (CR)	Average Variance Extracted (AVE)
Pro-environmental Attitudes	PEA	0.668–0.859	0.894	0.629
Subjective Norms	SN	0.808–0.885	0.881	0.712
Perceived Behavioral Control	PBC	0.758–0.841	0.844	0.644
Intention to Engage in PEB	Intention	0.555–0.880	0.896	0.594
Pro-environmental Behavior	PEB	0.421–0.753	0.851	0.528
Pro-environmental Innovative Behavior	IB	0.585–0.756	0.892	0.481
Sense of School Belonging	SB	0.452–0.744	0.923	0.512

**Table 3:** Statistical results of the hypothesized paths.

Hypothesis	Path	*B*	*SE*	*β*	*t*	*p*
H1a	PEA → IPB	0.952	0.039	0.921	24.12	<0.001
H1b	SN → IPB	0.272	0.021	0.305	12.85	<0.001
H1c	PBC → IPB	0.485	0.027	0.514	18.22	<0.001
H1d	IPB → PEB	0.638	0.022	0.681	28.5	<0.001
H2a	IB → PEA	0.198	0.021	0.219	9.45	<0.001
H2b	IB → SN	0.465	0.026	0.486	17.65	<0.001
H2c	IB → PBC	0.245	0.022	0.271	11.2	<0.001
H3a	SB → PEA	0.185	0.021	0.205	8.85	<0.001
H3b	SB → SN	0.382	0.026	0.402	14.9	<0.001
H3c	SB → PBC	0.188	0.021	0.208	9.15	<0.001
H3d	SB → IB	0.342	0.022	0.379	15.3	<0.001

Note. PEA = Pro-environmental attitudes; SN = Subjective norms; PBC = Perceived behavioral control; IPB = Intention to engage in pro-environmental behavior; PEB = Pro-environmental behavior; IB = Pro-environmental innovative behavior; SB = Sense of school belonging.

**Table 4:** Multigroup analysis by gender.

Hypothesis	Path	*β* (Female)	*p* (F)	*β* (Male)	*p* (M)	Path Difference
H1a	PEA → IPB	0.885	<0.001	0.945	<0.001	Stronger in males
H1b	SN → IPB	0.282	<0.001	0.32	<0.001	Comparable
H1c	PBC → IPB	0.49	<0.001	0.525	<0.001	Comparable
H1d	IPB → PEB	0.655	<0.001	0.705	<0.001	Stronger in males
H2a	IB → PEA	0.205	<0.001	0.23	<0.001	Comparable
H2b	IB → SN	0.455	<0.001	0.505	<0.001	Comparable
H2c	IB → PBC	0.255	<0.001	0.285	<0.001	Comparable
H3a	SB → PEA	0.18	<0.001	0.225	<0.001	Stronger in males
H3b	SB → SN	0.375	<0.001	0.42	<0.001	Comparable
H3c	SB → PBC	0.19	0.003	0.225	<0.001	Stronger in males
H3d	SB → IB	0.355	<0.001	0.4	<0.001	Comparable

Note. *β* represents standardized path coefficients. PEA = Pro-environmental attitudes; SN = Subjective norms; PBC = Perceived behavioral control; IPB = Intention to engage in pro-environmental behavior; PEB = Pro-environmental behavior; IB = Pro-environmental innovative behavior; SB = Sense of school belonging.

## Data Availability

The data presented in this study are available on request from the corresponding author.
